# Highly efficient SERS-based detection of cerebrospinal fluid neopterin as a diagnostic marker of bacterial infection

**DOI:** 10.1007/s00216-016-9535-7

**Published:** 2016-04-16

**Authors:** Agnieszka Kamińska, Evelin Witkowska, Aneta Kowalska, Anna Skoczyńska, Iwona Gawryszewska, Elżbieta Guziewicz, Dymitr Snigurenko, Jacek Waluk

**Affiliations:** Institute of Physical Chemistry, Polish Academy of Sciences, Kasprzaka 44/52, 01-224 Warsaw, Poland; National Medicines Institute, Chełmska 30/34, 00-725 Warsaw, Poland; Institute of Physics, Polish Academy of Sciences, Al. Lotników 32/46, 02-668 Warsaw, Poland; Faculty of Mathematics and Natural Sciences, College of Science, Cardinal Stefan Wyszyński University, Dewajtis 5, 01-815 Warsaw, Poland

**Keywords:** Surface-enhanced Raman spectroscopy (SERS), Neopterin, Cerebrospinal fluid, Bacterial infections, *Neisseria meningitidis*

## Abstract

A highly efficient recognition unit based on surface-enhanced Raman spectroscopy (SERS) was developed as a promising, fast, and sensitive tool for detection of meningococcal meningitis, which is an extremely serious and often fatal disease of the nervous system (an inflammation of the lining around the brain and spinal cord). The results of this study confirmed that there were specific differences in SERS spectra between cerebrospinal fluid (CSF) samples infected by *Neisseria meningitidis* and the normal CSF, suggesting a potential role for neopterin in meningococcal meningitis detection and screening applications. To estimate the best performance of neopterin as a marker of bacterial infection, principal component analysis (PCA) was performed in a selected region (640–720 cm^−1^) where the most prominent SERS peak at 695 cm^−1^ arising from neopterin was observed. The calculated specificity of 95 % and sensitivity of 98 % clearly indicate the effective diagnostic efficiency for differentiation between infected and control samples. Additionally, the limit of detection (LOD) of neopterin in CSF clinical samples was estimated. The level of neopterin was significantly higher in CSF samples infected by *N. meningitidis* (48 nmol/L), compared to the normal (control) group (4.3 nmol/L). Additionally, this work presents a new type of SERS-active nanostructure, based on polymer mats, that allows simultaneous filtration, immobilization, and enhancement of the Raman signal, enabling detection of spectra from single bacterial cells of *N. meningitidis* present in CSF samples. This provides a new possibility for fast and easy detection of bacteria in CSF and other clinical body fluids on a time scale of seconds. This method of detection produces consistent results faster and cheaper than traditional laboratory techniques, demonstrates the powerful potential of SERS for detection of disease, and shows the viability of future development in healthcare applications.

**Electronic supplementary material:**

The online version of this article (doi:10.1007/s00216-016-9535-7) contains supplementary material, which is available to authorized users.

## Introduction

Neopterin (2-amino-4-hydroxy-6-(d-*erythro*-1′,2′,3′-trihydroxypropyl)pteridine) is produced from guanosine triphosphate by human monocytes and macrophages after stimulation by interferon gamma (IFN-γ) derived from antigen-activated T lymphocytes [1, 2]. After activation of the immune system the level of neopterin in human body fluids is significantly increased. Thus, determination of neopterin may indicate the state of activation of the cellular immune system during subsequent stages of various diseases, such as rheumatoid arthritis (RA) [3], neuropsychiatric abnormalities [4], cardiovascular disease [5], insulin resistance [6], allograft rejection, and some tumors [7]. Elevated neopterin levels were also observed in viral infections [8–11] (hepatitis A, B, and C, cytomegalovirus, measles, rubella, influenza), and bacterial infections [12]. In patients with sepsis (a consequence of metabolic and hemodynamic events caused by microbial invasion), plasma concentrations of neopterin are increased compared with healthy controls [13].

Bacterial meningitis might be associated with both, elevated serum and cerebrospinal fluid (CSF) neopterin levels compared to controls [10, 14, 15]. In brucellosis, neopterin levels were a mean of 52.5 mmol/mL, significantly higher than for healthy controls (<5 nmol/L) and patients with tuberculosis [16]. In leprosy caused by *Mycobacterium leprae,* 75 % of patients with tuberculoid and lepromatous leprosy presented elevated urinary neopterin excretion [17].

In the context of rising drug resistance and difficulties in monitoring drug compliance, a new diagnostics marker needs to be explored. It may also be useful to distinguish active forms of disease from latent ones. Within the group of bacterial infections it was shown that patients with symptoms lasting for at least 5 days had significantly higher neopterin concentrations than patients with acute illness. Investigations on critically ill patients in intensive care units evaluated neopterin levels as a tool to discriminate patients with systemic inflammatory response syndrome with and without infectious etiology. Neopterin levels were found to have a specificity of 78 % for discriminating infectious and noninfectious etiology of critical illness [18].

The measurement of the neopterin levels can provide reliable information regarding the disease diagnosis, stage, prognosis, and is also important for monitoring the response to therapy. Screening of neopterin concentrations in blood donations allows one to detect acute infections in a nonspecific way and improves safety of blood transfusions. Up to now several analytical procedures have been applied for evaluation of the neopterin level in blood using mainly high pressure liquid chromatography (HPLC) [19] and enzyme-linked immunosorbent assay (ELISA) [20] techniques.

However, both of these methods are effortful and expensive, and moreover they require technical equipment and highly qualified personnel. Additionally, the difficulties and inaccuracy of existing neopterin assays have been presented, and a number of factors have been shown to affect the validity and quality of such measurements [21–23]. To the best of our knowledge, all immunoassays which are used for this immune marker are at or near their limit of measurement. Therefore, there is a need to develop a more sensitive, selective, stable, and durable method for the specified biomarkers.

As was mentioned, the main method for determination of the selected immune markers relies heavily on various ELISA kits. Alternative approaches during recent decades have usually used fluorescent antibody assays, evanescent wave interference, and electrochemical methods. Current successes in nanotechnology and instrumentation development have led to recognition of biomolecular systems based on surface-enhanced Raman spectroscopy (SERS) with a higher sensitivity and a more clear visualization of bioanalytes. In brief, surface-enhanced Raman scattering is a vibrational spectroscopy that relies on enhancement of the electromagnetic field due to resonance between the excitation light and surface plasmons of the SERS nanostructures [24, 25]. This electromagnetic effect is the main contributor in SERS enhancement and may increase the Raman signal up to 10^11^-fold. A chemical mechanism is also believed to take place in SERS enhancement owing to the charge transfer between adsorbed molecules and the metal conduction band of metallic nanostructures. This chemical mechanism may provide an enhancement factor (EF) of less than 4 orders of magnitude [26, 27]. Both these mechanisms ensure enhancement of Raman signal with single molecule resolution [28]. Another interesting point about SERS is the linear dependence of SERS intensity on the power of incident light despite the extraordinary nonlinear effect of signal enhancement. Therefore, SERS technology can be used for the quantitative measurement of analytes with ultrahigh sensitivity [29]. The SERS technique therefore extends the range of Raman applications to more sensitive, specific, and fast detection of a wide range of analytes, e.g., nucleic acids and proteins [30], therapeutic agents [31], drugs and trace materials [32], microorganisms [33], and cells [34].

In this study we present our research efforts aimed at detection of specific bacterial infection caused by *Neisseria meningitidis* using surface-enhanced Raman spectroscopy. Anton Weichselbaum first isolated *N. meningitidis* from the CSF of a patient [35]. This bacterium is a Gram-negative diplococcus and belongs to pathogenic members of the *Neisseriaceae* family [36]*. N. meningitidis* is one of the three main bacteria that cause acute bacterial meningitis, along with *Streptococcus pneumoniae* and *Haemophilus influenzae* [37].

*N. meningitidis* only infects humans and the average incubation period is 4 days, but it can range between 2 and 10 days. Meningitis caused by this bacteria is usually very serious (5 % to 10 % of patients die, typically within 24 to 48 h after the onset of symptoms) and requires rapid detection and urgent medical attention with appropriate antibiotic therapy. Taking into account the high mortality rates, rapid detection of bacteria in CSF and subsequent effective treatment are essential.

In this work we present also, for the first time, the possibility of using neopterin levels for diagnosis of meningococcal meningitis disease in CSF clinical samples already diagnosed by microbiological techniques. Additionally, the obtained results were compared with ELISA as the reference method. We use SERS combined with a multivariate statistical method (principal component analysis, PCA) to differentiate between CSF control clinical samples (from healthy patients) and CSF clinical samples infected by *N. meningitidis*. Moreover, besides the Si/ZnO/Au platform used for neopterin level calculation, a new SERS substrate based on a polymer mat was applied for simultaneous filtration, immobilization, and enhancement of the Raman signal for the detection of single bacterial cells.

## Materials and methods

### Chemicals and materials

Neopterin was purchased from Tocris Bioscience (Bristol, UK). Water (resistivity over 18 MΩ cm) was purified using a Milli-Q plus 185 system and used in all experiments. The CSF and *N. meningitidis* strain were obtained from the National Reference Centre for Bacterial Meningitis (NRCBM) in the National Medicines Institute (NMI) in Warsaw. The neopterin levels in CSF were estimated by a commercial ELISA test (IBL International GmbH, Hamburg).

### Instrumentation and data collection

Raman and SERS spectra of analyzed samples were recorded using the Renishaw inVia Raman system with 1024 × 256 pixel Peltier-cooled RenCam CCD detector. All measurement were performed using ×20 microscope objective (numerical aperture = 0.25), focusing the 785-nm laser to a spot size of approximately 5 μm. SERS spectra were acquired from less than 5 mW of incident laser power at ambient conditions using a back-scattering geometry. The SERS spectra were recorded between 400 and 1600 cm^−1^ at resolution of ca. 2 cm^−1^. The typical acquisition time was 10 s for a single SERS measurement. The obtained spectra were processed with OPUS software provided by Bruker. All spectra were smoothed, baseline corrected, and normalized.

SEM measurements were conducted using the FEI Nova NanoSEM 450 with an accelerating voltage of 10 kV under high vacuum.

### SERS nanostructures fabrication

#### Si/ZnO layers

Atomic layer deposition (ALD) was used for zinc oxide layer deposition on Si(100) at 100 °C. Diethylzinc and deionized water were used as precursors, and nitrogen was used as purging gas [29]. The process was conducted in the Savannah-100 reactor. Typically the ZnO layers were grown with 10,000 ALD cycles, which lead to approximately 1.4 μm thickness.

#### Electrospun polymer mats

The poly(l-lactide) (PLA) mats were purchased from MECC Co., Ltd., Japan and cut into squares (area of 0.25 cm^2^).

#### Procedure for SERS nanostructure gold sputtering

Si/ZnO layers and electrospun polymer mats were covered with a thin layer of sputtered gold (ca. 90 nm) using PVD equipment from Leica (model EM MED020). Au target was obtained from Mennica Metale Szlachetne, Warsaw, Poland. During this procedure a vacuum level of 10^−2^ mbar and current of 25 mA were applied.

### Bacterial sample preparation for microbiological and SERS experiments

Clinical CSF samples were obtained as a part of routine activity of the NRCBM and were analyzed anonymously. All the data were collected in accordance with the European Parliament and Council decision for the epidemiological surveillance and control of communicable disease in the European Community [38, 39]. Thus ethical approval and informed consent were not required. *N. meningitidis* of serogroup B (603/2011) used during the study serves as a reference strain in polymerase chain reaction (PCR) in the NRCBM.

### Microbiological confirmation of *N. meningitidis*

In the case of negative culture, the NRCBM has been receiving clinical materials, including CSF, from patients with suspected invasive meningococcal disease. The DNA isolated from these samples was used for PCR to identify *N. meningitidis* [40, 41].

The strain was identified on the basis of typical morphology of colonies, Gram stain, oxidase test, and API NH test (bioMerieux, Marcy-l’Etoile, France) according to the manufacturer’s instructions. Serogroup was determined by slide agglutination tests using commercial antisera (Remel).

### Bacterial culture and SERS sample preparation

*Neisseria meningitidis* used in the experiment was obtained from the NRCBM in Warsaw, Poland. To multiply microbial organisms, we cultivated them on solid BHI (brain heart infusion) growth medium at 37 °C for 24 h. After that, some bacterial colonies were redispersed in saline solution (sterile 0.9 % NaCl solution) and centrifuged for 5 min at 4000 rpm (so as not to destroy the cell membrane). The centrifugation process in the fresh saline solution was repeated four times to obtain a solution of clean bacterial cells, at a concentration of *N. meningitidis* of 10^6^ CFU/mL. The density of bacterial cells was determined by counting the amount of colonies which had grown on the Petri dish from a known amount of medium. The count was taken after 1 day of cultivation at 37 °C. Before carrying out Raman measurements, 10 μL of an aqueous bacterial solution was placed over the SERS substrate. Measurements were taken after 5 min.

### Chemometrics

The SERS spectra were prepared for PCA using a two-step approach. First, OPUS software (Bruker Optic GmbH 2012 version) was used to smooth the spectra with the Savitsky–Golay filter, the background was removed using baseline correction, and then the spectra were normalized using a so-called Min-Max normalization (the area of band around 963 cm^−1^). All the data were transferred to the Unscrambler software (CAMO software AS, version 10.3, Norway) where PCA was performed. PCA is a multivariate technique that reduces the dimensionality of complex spectroscopic data from many wavenumber assignments to a few principal components (PCs), making it easier to identify the majority of variations within the spectra [42].

PCA reduces the complexity of high-dimensional SERS data from many wavenumber assignments to several PCs. Each PC represents a linear combination of the original variables (e.g., Raman wavenumber). The first component (in the horizontal direction) is the most important one and accounts for as much variation in the data as possible. In the PCA model, the big spectral set matrix (**X**) is transformed into two smaller matrices according to the formula **X** = **TP**^T^ + **E** where **T** is the matrix of scores, **P** is the matrix of loadings, and **E** is the error matrix. The PCA method enables one to understand the sources of variation in the obtained SERS data, e.g., the plot of loadings vs. the wavenumber indicates the most important diagnostic vibrations in the spectra.

In our study, PCA was carried on two different data sets, consisting of spectra obtained for normal CSF (without microbiological confirmation of *N. meningitidis* infection) and for CSF infected by this pathogen. Additionally, the obtained data allow one to calculate the diagnostic sensitivity and specificity according to the formula [43]

$$ \begin{array}{l}\mathrm{Sensitivity}=\mathrm{T}\mathrm{P}/\left(\mathrm{T}\mathrm{P}+\mathrm{F}\mathrm{N}\right)\hfill \\ {}\mathrm{Specificity}=\mathrm{T}\mathrm{N}/\left(\mathrm{T}\mathrm{N}+\mathrm{F}\mathrm{P}\right)\hfill \end{array} $$

where TP and TN are the numbers of true positive and true negative results, respectively; FN and FP are the numbers of false negative and false positive results, respectively.

## Results and discussion

### SERS analysis of CSF and neopterin: comparison between normal healthy control and *N. meningitidis-*infected clinical samples

**Fig. 1:**
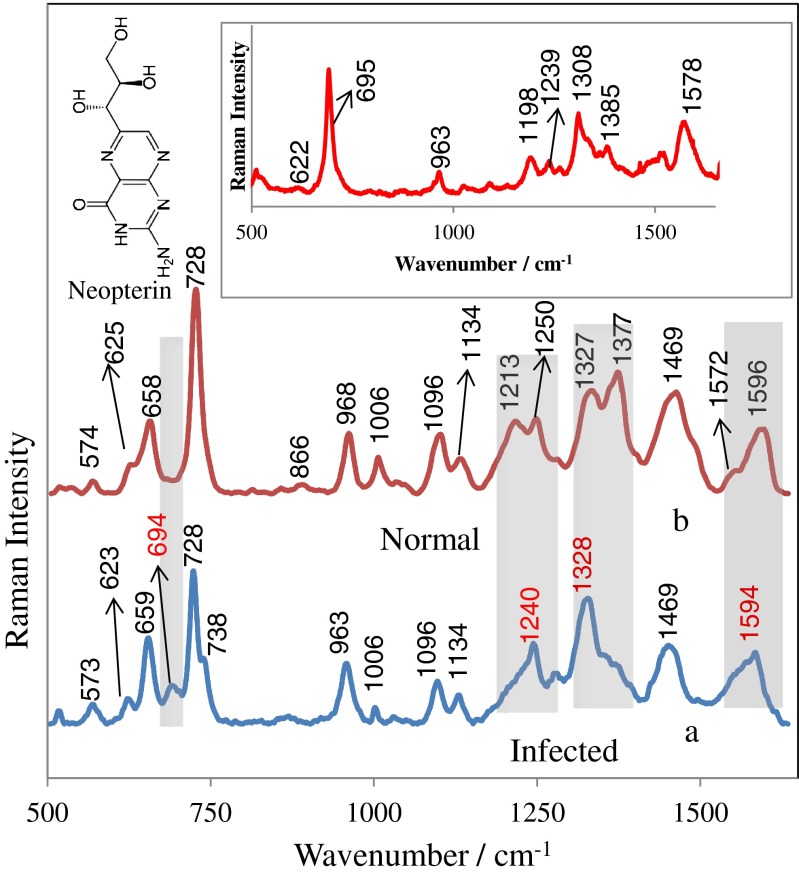
Comparison of the SERS spectrum of the CSF samples infected by *N. meningitidis* (**a**) versus that of the normal (control) CSF samples (**b**). Samples of CSF were deposited onto the Si/ZnO/Au substrate and measured in situ. The *inset* shows the SERS spectrum of neopterin adsorbed onto the Si/ZnO/Au substrate from 35.0 nmol/L neopterin solution in a PBS buffer. The presented SERS spectra were averaged from ten measurements in different places of the SERS nanostructures

**Fig. 2:**
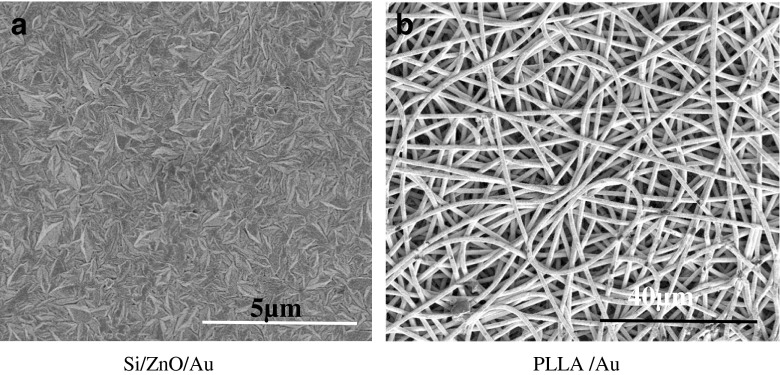
SEM images of the SERS nanostructures: (**a**) Au-coated Si/ZnO and (**b**) PLA polymer mat

Neopterin appears in human CSF, blood (plasma, serum), and urine, and its increased levels indicate activation of the immune system involved in the pathogenesis and/or affected by malignant diseases. An earlier literature report [44] reveals that CSF neopterin characterization at different stages of HIV infection can serve as a useful method for the diagnosis of this disease and in monitoring the central nervous system inflammatory effects; it can also give valuable information regarding the cause of ongoing brain pathology. Our results showed that neopterin is biologically and chemically stable as a biomarker in human biological fluids, gives very strong SERS signals, and it can be easily quantified using SERS. The inset in Fig. 1 presents the SERS spectrum of neopterin adsorbed onto the Si/ZnO/Au substrate from 35.0 nmol/L neopterin solution in a PBS buffer. The morphological structure of the Si/ZnO/Au platform is presented in Fig. 2. The SERS spectrum of neopterin is dominated by bands at 695, 1308, and 1578 cm^−1^ due to C–C vibration and ring modes, N–H bending modes, and NH_2_ symmetric deformation, respectively [45]. In this work we examine the CSF neopterin changes in bacterial infections. SERS characterization of CSF samples of both healthy subjects and patients with confirmed bacterial meningitis was carried out. Figure 1 shows the normalized SERS spectra of CSF samples infected by *N. meningitidis* (1a) and the normal (control) CSF samples (1b) deposited onto the Si/ZnO/Au substrate. CSF of the healthy patient contains no erythrocytes and up to five leukocytes per microliter [46]. The intense bands at 728, 1096, 1134, 1377, and 1596 cm^−1^ were assigned to vibrations of the nucleic bases of DNA and lipids [47]. The features at 1469, 1250, 1006, and 964 cm^−1^ are associated with CH_2_ deformation, the amide III, the symmetric ring breathing bands of phenylalanine and protein, and C–C stretching, respectively [48]. Aromatic amino acid residues, phenylalanine, tyrosine, and tryptophan were expected to have bands at 625, 659, 866, 1173, and 1213 cm^−1^. The prominent SERS peaks located at 659, 728, 963, 1006, 1096, 1134, and 1469 cm^−1^ can also be consistently observed in infected samples (Fig. 1b). However, a detailed analysis of SERS spectra of CSF from patients affected by bacterial meningitis reveals remarkable differences. The new SERS band at 695 cm^−1^ which corresponds to the C–C vibration and ring modes of neopterin [49] can be observed only in the infected samples and is absent in healthy subjects. This indicates the increased contribution of neopterin in bacterial infections. In the range 500–1600 cm^−1^, SERS spectra of CSF are complex and provide a rich source of information about numerous CSF components. The region of CSF (660–720 cm^−1^) in which the marker band of neopterin appears (695 cm^−1^) is devoid of other intensive vibrations and allows label-free analysis of neopterin. There are also distinctive SERS features and intensity differences for normal and infected CSF samples in the spectral ranges of 1200–1260, 1270–1380, and 1500–1650 cm^−1^, indicated by the gray-shaded columns in Fig. 1. These differences in the SERS spectral pattern could reflect changes in the quantity and structure of proteins and peptides of CSF associated with abnormal metabolism of patients with bacterial infections. For example, in infected samples the bands at 1213 and 1250 cm^−1^ due to the C–H in-plane bending of phenylalanine and/or tyrosine and amide III band of proteins (α-helix) disappear and a new band at around 1240 cm^−1^ is observed, indicating transition from an α-helical to β-sheet structure [50] of certain proteins in infected CSF samples. Moreover, the SERS band of tryptophan at 1572 cm^−1^ was found to be higher in normal CSF samples, suggesting a decrease in the relative amount of tryptophan and also larger amount of neutral amino acids like phenylalanine, histidine, and isoleucine [51] in CSF with *N. meningitidis.* It should be highlighted that these changes in the three regions mentioned above appear only in real clinical samples of CSF infected by *N. meningitidis* bacterium. Figure S1 in the Electronic Supplementary Material (ESM) shows the comparison of a SERS spectrum for a clinical sample of CSF infected by *N. meningitidis* (b) versus that of normal CSF with added 15 nmol/L solution of neopterin (a). As can be seen, for samples of CSF with neopterin “artificially” inserted (ESM Fig. S1) only the marker band at 659 cm^−1^ corresponding to neopterin appears. The SERS differences that indicate the changes in quantity and structure of proteins and peptides of CSF (changes in the regions of 1200–1260, 1270–1380, and 1500–1650 cm^−1^) were not observed. This is clear evidence that these spectral features are correlated only with anomalous metabolism of patients with bacterial infection.

### Chemometrics analysis

Although significant spectral differences between normal and infected CSF have been clearly observed in SERS spectra, this simplistic analysis of the experimental observations uses only limited number of SERS peaks or SERS regions. Therefore, multivariate statistical analysis in the form of PCA has been exploited to utilize the whole spectra and to automatically determine the diagnostic marker bands for improving the efficiency of CSF diagnosis and also to find out the statistical significance of the proposed method. This analysis method is known as an excellent approach towards reducing dimensionality of Raman data and is widely used by researchers in discriminating cancer tissues from that of a control subject [52], for identification of pathogens in body fluids [53], and for blood analysis [49].

**Fig. 3:**
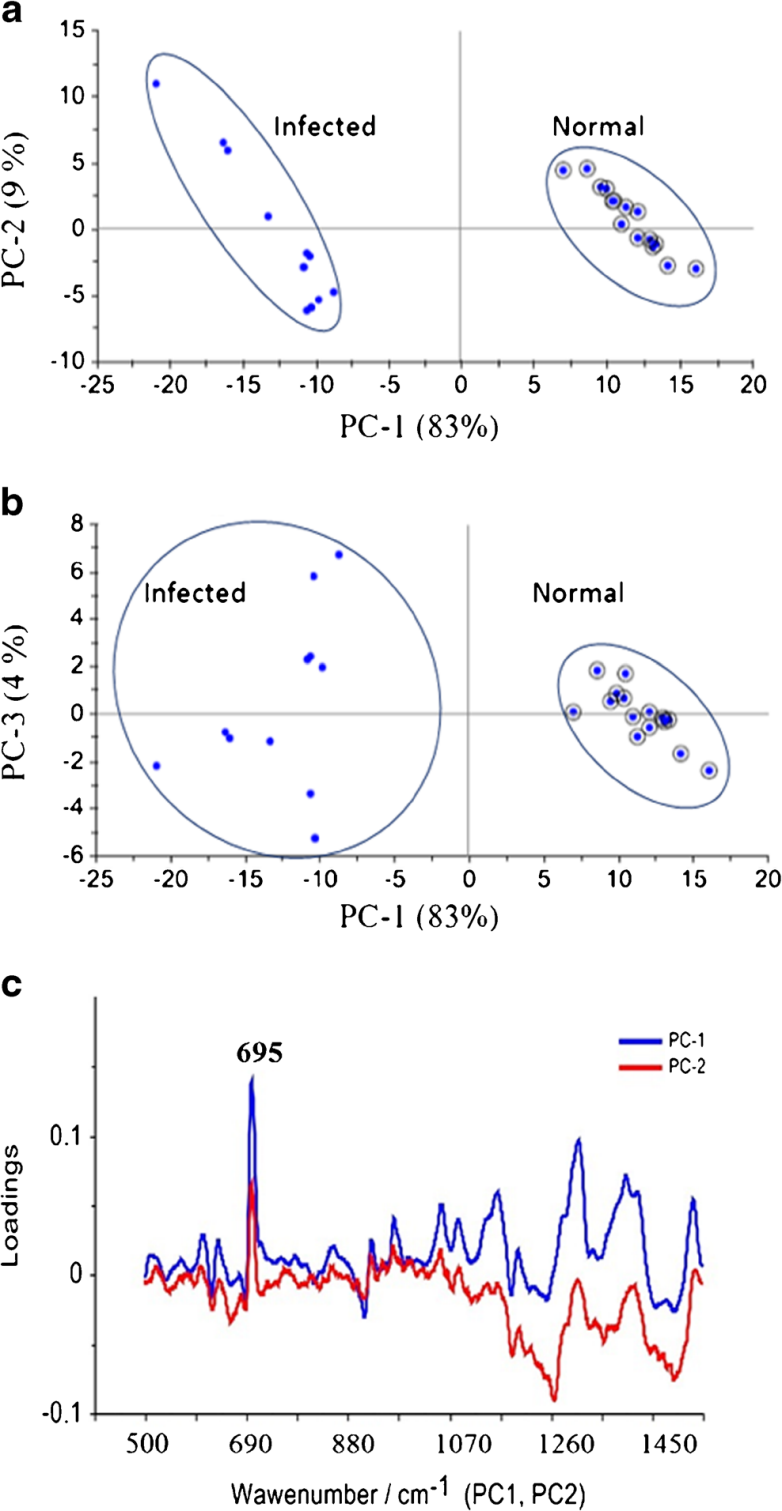
(**a**) Plot of the first principal component PC1 (83 % of the total variance) versus the second principal component PC2 (9 % of the total variance) and (**b**) plot of the first principal component PC1 (83 % of the total variance) versus the third principal component PC3 (4 % of the total variance) for the normal group versus infected group. (**c**) Loadings plots of the first and second principal components PC1 and PC2, highlighting regions associated with large loadings

The PCs are used to build a model with a resolution of recognition. Initially, the analysis was performed in the whole spectral region between 500 and 1650 cm^−1^. In the first step we found that three PCs (PC1, PC2, PC3) are the most diagnostically significant and explain 94 % of the variance in the data. The resulting PC scores plots (Fig. 3a, b) indicate diagnostic utility to differentiate between analyzed samples. As can be seen, it is possible to separate the normal CSF data points and *N. meningitidis*-infected CSF data points along the axis of the first PC.

The loadings of the PCs provide information on the variables (wavenumber of the spectrum) that are important for group separation. Figure 3c displays the loadings plots of PC1 and PC2 for the whole wavenumber region 500–1600 cm^−1^. By analysing these plots one can indicate the most important diagnostic variables in the analyzed data set. Variables with high loading values are the most important for diagnostic purposes. The loadings of both PC1 and PC2 exhibit a positive peak attributed to the main marker bands of the neopterin (Fig. 3c). That is why, for the best classification among the two groups of analyzed samples, the PCA was performed for the selected narrow region (640–720 cm^−1^) where the most prominent SERS peak at 695 cm^−1^ arising from neopterin is observed (see Fig. 1b and inset in Fig. 1).

**Fig. 4:**
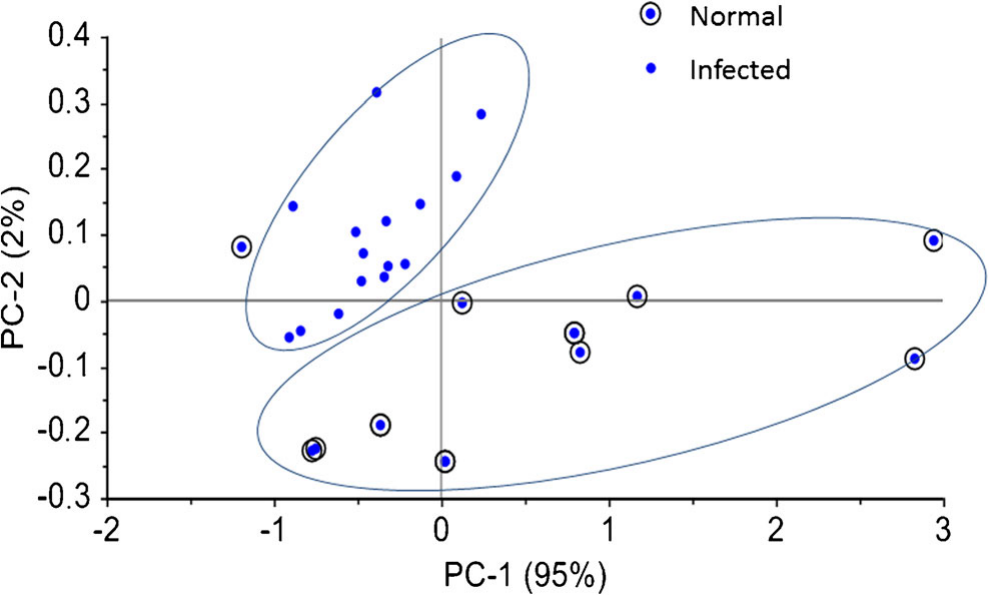
Scatter plots of the first two principal components PC1 (95 % of the total variance) and PC2 (2 % of the total variance) of the scores belonging to the control and *N. meningitidis*-infected CSF categories for the 640–720 cm^−1^ diagnostic range

Figure 4 displays the score plot of the first two principal components PC1 (95 % of the total variance) and PC2 (2 % of the total variance) of the normal CSF and *N. meningitidis*-infected CSF SERS spectra. The stepwise analysis in this area gives a more effective diagnostic efficiency for distinguishing between infected and control samples. A specificity of 98 % and sensitivity of 95 % were obtained. These results demonstrate that the marker band of neopterin (695 cm^−1^) can be used for meningococcal meningitis detection in CSF samples.

### Measurement of neopterin concentration in CSF

**Fig. 5:**
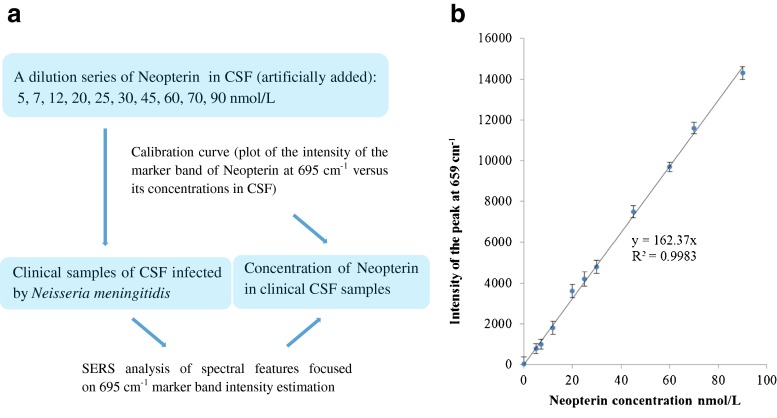
(**a**) Scheme of the detection mechanism of neopterin. (**b**) The plot of the marker band intensity (695 cm^−1^) versus the neopterin concentration in CSF. The *error bars* indicate the standard deviations from ten averaged measurements

The measurement of neopterin concentration in human body fluids such as serum, urine pleural fluid, and CSF provides an insight into cell-mediated immune responses. In this study we also examined the neopterin levels in samples infected by *N. meningitidis* and in healthy controls using the SERS technique. Figure 5a presents the scheme of the detection mechanism of neopterin applied in this experiment. The calibration curve was built on the basis of the plot of SERS intensity (band at 695 cm^−1^) versus the concentration of neopterin in normal CSF (Fig. 5b). The neopterin was artificially added to normal CSF samples in clinically important concentrations (0.0–90.0 nmol/L). The calibration curve was fitted over the analyzed region (*y* = 162.37*x*) and the correlation coefficient of 0.998 was calculated. The linear dependence of the SERS intensity at 695 cm^−1^ (*y*) on the neopterin concentration (*x*) in the whole examined region was evident. Moreover, the limit of detection (LOD = 1.6 nmol/L) was assessed on the basis of the signal-to-noise method [54]. This calibration line was used to estimate the concentrations of neopterin in clinical samples of CSF infected by *N. meningitidis*. The neopterin concentrations of the same samples were also evaluated using a commercial ELISA kit (IBL International). Furthermore, the results were evaluated by comparing with control samples and estimated values are described in Table 1.

**Table 1:** CSF neopterin concentration obtained using two methods, SERS and ELISA

	SERS (new method)	ELISA (reference method)
Control CSF (nmol/L)	3.8 ± 0.7	4.0 ± 1.3
Infected CSF (nmol/L) (with *Neisseria meningitidis)*	30.0 ± 4.1	36.0 ± 5.2

It should be highlighted that for normal healthy control samples of CSF the level of neopterin was below 4.3 nmol/L. A value below 5 nmol/L is a typical CSF neopterin concentration calculated for patients without a modified immune system [14]. In contrast, CSF neopterin concentrations determined by radioimmunoassay (RIA) were 63.0 nmol/L for patients with acute bacterial meningitis, 32.5 nmol/L for patients with Lyme neuroborreliosis, and 130.9 nmol/L in individuals with viral meningitis [14].

These results clearly demonstrate that neopterin may be used as a marker in the meningococcal meningitis diagnosis and monitoring of this infection.

### Detection of *N. meningitidis* in CSF

**Fig. 6:**
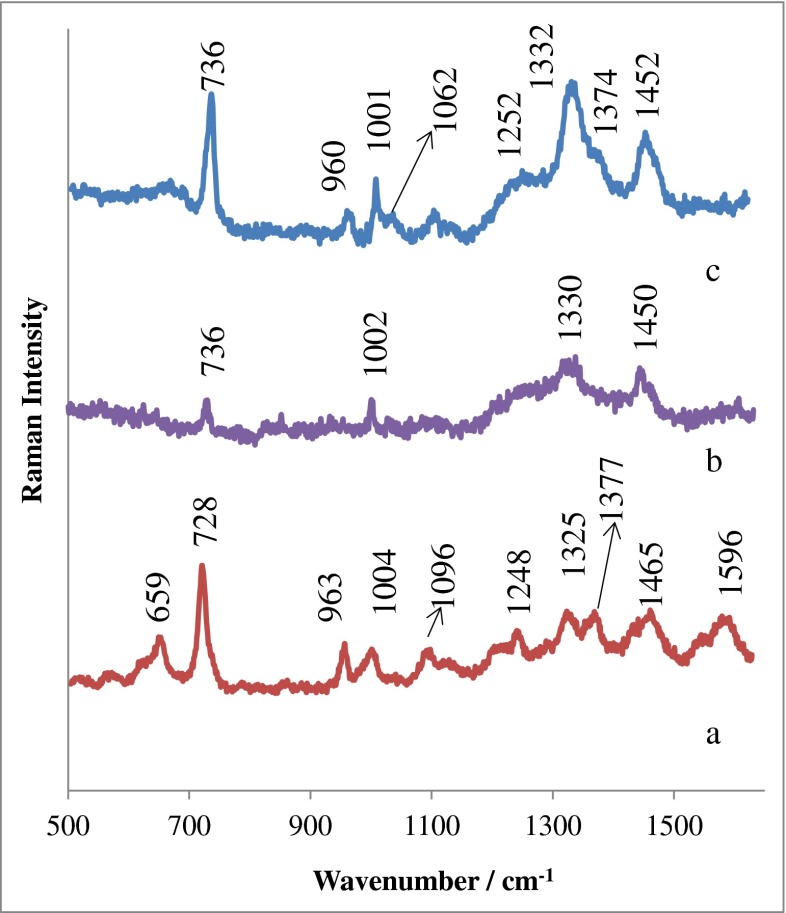
The SERS spectrum of (**a**) CSF components trapped on a polymer mat-based SERS platform, (**b**) the remaining part of the filtrated solution deposited again on the Si/ZnO/Au substrate, and (**c**) the SERS spectrum of *N. meningitidis* multiplied before SERS experiment by cultivation in liquid LB (Lysogeny broth) growth medium and then deposited onto the Si/ZnO/Au substrate

Surface-enhanced Raman spectroscopy is a powerful technique for the rapid and accurate detection of pathogenic bacteria in biological fluids [33]. In our laboratory, we have developed a new type of SERS nanostructure that permits the simultaneous filtration of bacteria from solutions, immobilization onto the SERS nanostructures, and their Raman signals enhancement. The applied SERS substrate is based on a gold-covered electrospun polymer mat [55]. The morphology of this SERS substrate is presented in Fig. 2. In the present study we used this SERS substrate for detection of *N. meningitidis* in CSF samples. The applied technique is simple, fast, and allows filtration and/or concentration of CSF composites (leukocytes, albumins, globulins, and bacteria) within a small area of the SERS nanostructures. The setup for the proposed method consists of a Büchner flask, a filter funnel, and a vacuum pump [55]. The detection of bacteria was performed in two steps. In the first step, a single droplet of infected CSF was placed on the SERS platform positioned on a Büchner flask. The vacuum pump caused the movement of the fluid through SERS-active polymer mat. In the next step, several droplets of a PBS buffer solution (at about 0.3 μL) were passed through the polymer mat in the same way. Taking into account the size of pores in the polymer mat (about 3–5 μm) and the size of CSF components (leukocytes from 10 up to 15 μm [56], albumins from 3 to 10 nm [57]), and the size of *N. meningitidis* bacterium, which ranges from 0.6 to 1.0 μm [58]*,* we are able to separate these species. The largest leukocytes (the main component of CSF) remain on the polymer mat, while the smaller proteins and bacteria pass through. Figure 6 shows the SERS spectra of CSF infected with *N. meningitidis* after filtration on the SERS polymer platform. Figure 6a present the SERS spectrum of CSF components trapped on the SERS platform. The observed spectral features are characteristic for leukocytes (Sect. “Measurement of neopterin concentration in CSF” and Fig. 2). Figure 6b depicts the SERS spectrum of the remaining part of the filtrated solution deposited again on the Si/ZnO/Au substrate. The Raman spectrum of *N. meningitidis* has not been presented in the literature; therefore we attempted, for the first time, the SERS measurements and band assignments of this bacterium. Figure 6c presents the SERS spectrum of *N. meningitidis* multiplied before SERS experiment by cultivation in a liquid LB (Lysogeny broth) growth medium and then deposited on the Si/ZnO/Au substrate. As can be seen in Fig. 6c, the intense band at about 736 cm^−1^ appears in the SERS spectrum of *N. meningitidis*. This band is observed in many types of bacterial species like *Escherichia coli, Salmonella enterica, Staphylococcus epidermidis, Staphylococcus aureus*, and *Bacillus megaterium* [33, 55]. This band is due to the C–N stretching mode vibration of the adenine of lipid layer in the cell wall or to the breathing mode of the purine ring [59, 60]. Jarvis et al. assigned this band to the vibration of the glycosidic ring mode from cell wall components [61]. Typical Raman bands of proteins, phospholipids, and polysaccharides can be also observed in the SERS spectrum of *N. meningitidis*. The other observed bands can be attributed to amino acids (tyrosine, phenylalanine), or to C–C vibrations in phosphate binding of DNA, respectively [61].

The SERS spectrum of this bacterium also indicates peaks assigned to amide III (1252 cm^−1^) and CH_2_ vibrations (1450 cm^−1^) [59]. Table S1 (see ESM) shows band assignments for *N. meningitidis.* The most intense bands at 736, 1002, 1330, and 1450 cm^−1^ were also observed in the remaining part of a filtrated solution where the presence of, inter alia, bacteria is expected (Fig. 6b). As exemplified by Fig. 6, we are able to detect and identify the bacterial cell from CSF samples using our novel SERS substrates.

## Conclusions

This work presents a new label-free method for neopterin detection based on the SERS technique and the possibility of using this method for determination of the neopterin levels in CSF. It was also shown that CSF neopterin evaluation may be used in determining the bacterial meningitis infections caused by *N. meningitidis.* Additionally, a new class of SERS substrates based on a polymer mat was developed for simultaneous filtration, immobilization, and enhancement of the Raman signal, which allows the detection of single bacterial cells of *N. meningitidis* present in CSF samples. This opens a new route for simple identification of bacteria in CSF and other clinical body fluids on a time scale of seconds. In conclusion, neopterin emerges as an important biomarker and a strong predictor of bacterial meningitis infections. In the very near future, this study will be extended to a larger number of clinical samples to improve the diagnostic sensitivity and selectivity.

## Electronic supplementary material

Below is the link to the electronic supplementary material.

ESM 1 (PDF 70 kb)
