# Supplementary material for: Highly efficient SERS-based detection of cerebrospinal fluid neopterin as a diagnostic marker of bacterial infection
Source: Anal Bioanal Chem. 2016 Apr 16;408:4319–27. doi: 10.1007/s00216-016-9535-7 (PMC4875960; doi:10.1007/s00216-016-9535-7)
Supplement: Supplementary file 1 — (PDF 70 kb) [file 216_2016_9535_MOESM1_ESM.pdf]

Analytical and Bioanalytical Chemistry

Electronic Supplementary Material

Highly efficient SERS-based detection of cerebrospinal fluid neopterin as a diagnostic marker of bacterial infection

Agnieszka Kamińska, Evelin Witkowska, Aneta Kowalska, Anna Skoczyńska,
Iwona Gawryszewska, Elżbieta Guziewicz, Dmytro Snigurenko, Jacek Waluk

1. Comparison between the clinical sample of CSF and CSF with added neopterin

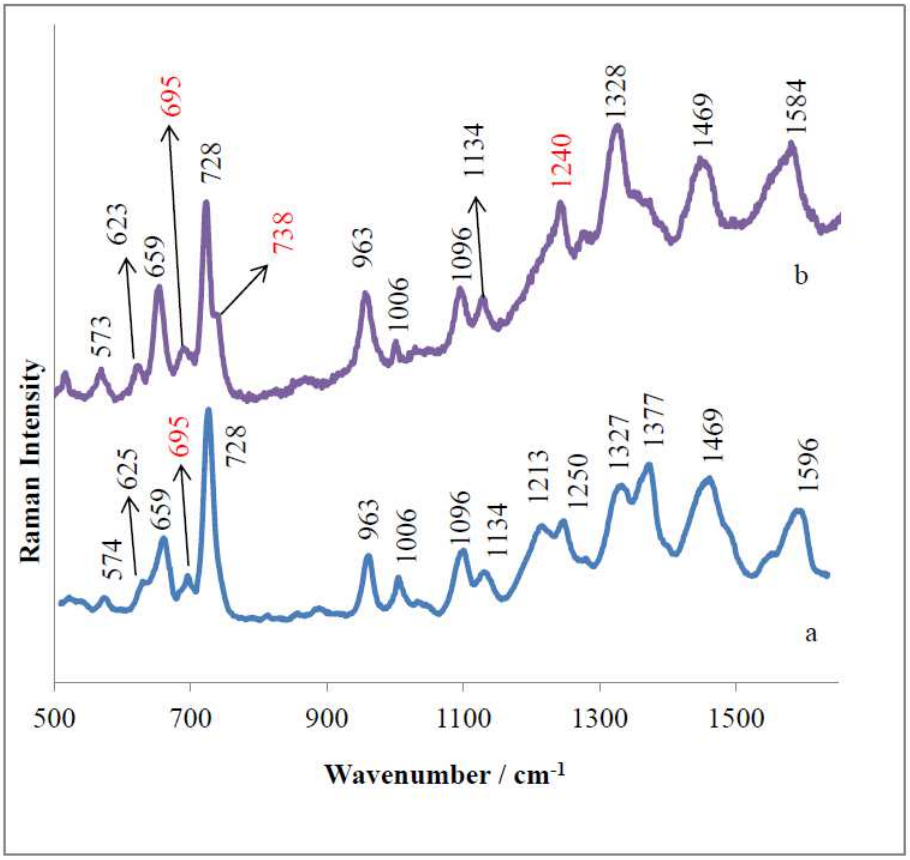

Fig. S1 Comparison of the SERS spectrum of a clinical sample of CSF infected by *Neisseria meningitidis* (b) with that of normal CSF with added 15 nmol/L solution of neopterin (a). Samples of CSF were deposited onto Au/ZnO/Si substrate and measured in situ. Experimental conditions: 5mW of 785 nm excitation, 4 x 10 seconds acquisition time. The SERS spectra have been shifted vertically for better visualization

Table S1 Band assignments for *Neisseria meningitidis* bacterium. The spectrum was acquired for Si/ZnO/ Au SERS substrate (data based on [68])

	Assignment	Wave number
1.	guanine, tyrosine	652
2.	Adenine	736
3.	C=C deformation	962
4.	phenylalanine	1001
5.	carbohydrates, mainly C-C (skeletal)	1062
6.	Amide III (random)	1252
7.	adenine, guanine, CH deformation	1332
8.	(COO–)	1374
9.	CH_2 deformation	1452
